# Nanoparticle-Based Delivery of *Anaplasma marginale* Membrane Proteins; VirB9-1 and VirB10 Produced in the *Pichia pastoris* Expression System

**DOI:** 10.3390/nano6110201

**Published:** 2016-11-05

**Authors:** Bing Zhang, Antonio S. Cavallaro, Karishma T. Mody, Jun Zhang, James R. Deringer, Wendy C. Brown, Timothy J. Mahony, Chengzhong Yu, Neena Mitter

**Affiliations:** 1 Department of Agriculture and Fisheries, Agri-Science Queensland, Animal Science, St Lucia, QLD 4072, Australia; bing.zhang@daf.qld.gov.au; 2 Queensland Alliance for Agriculture and Food Innovation, The University of Queensland, St Lucia, QLD 4072, Australia; a.cavallaro@uq.edu.au (A.S.C.); k.mody@uq.edu.au (K.T.M.); t.mahony@uq.edu.au (T.J.M.); 3 Australian Institute for Bioengineering and Nanotechnology, The University of Queensland, St Lucia, QLD 4072, Australia; jun.zhang1@uqconnect.edu.au (J.Z.); c.yu@uq.edu.au (C.Y.); 4 Department of Veterinary Microbiology and Pathology, Washington State University, Pullman, WA 99164, USA; jderinger@vetmed.wsu.edu (J.R.D.); wbrown@vetmed.wsu.edu (W.C.B.)

**Keywords:** *Anaplasma marginale*, VirB9-1, VirB10, *Pichia pastoris*, silica vesicles, vaccine adjuvants, immune responses

## Abstract

Bovine anaplasmosis or cattle-tick fever is a tick-borne haemolytic disease caused by the rickettsial haemoparasite *Anaplasma marginale* in tropical and subtropical areas of the world. While difficult to express, the proteins VirB9-1 and VirB10 are immunogenic components of the outer membrane type IV secretion system that have been identified as candidate antigens for vaccines targeting of *A. marginale*. Soluble VirB9-1 and VirB10 were successfully expressed using *Pichia pastoris*. When formulated with the self-adjuvanting silica vesicles, SV-100 (diameter: 50 nm, and pore entrance size: 6 nm), 200 µg of VirB9-1 and VirB10 were adsorbed per milligram of nanoparticle. The VirB9-1 and VirB10, SV-100 formulations were shown to induce higher antibody responses in mice compared to the QuilA formulations. Moreover, intracellular staining of selected cytokines demonstrated that both VirB9-1 and VirB10 formulations induced cell-mediated immune responses in mice. Importantly, the SV-100 VirB9-1 and VirB10 complexes were shown to specifically stimulate bovine T-cell linages derived from calves immunised with *A. marginale* outer membrane fractions, suggesting formulations will be useful for bovine immunisation and protection studies. Overall this study demonstrates the potential of self-adjuvanting silica vesicle formulations to address current deficiencies in vaccine delivery applications.

## 1. Introduction

*Anaplasma marginale*, an intra-erythrocytic gram negative bacterium, is a rickettsial haemoparasite of cattle, causing anaplasmosis or cattle-tick fever. The acute phase of this tick-borne disease is characterised by severe anaemia, weight loss, fever, abortion, lower milk production and often death [1]. After recovery from acute infection, cattle remain persistently infected without clinical signs, acting as reservoirs for transmission by ticks to other cattle [2]. The major surface proteins (MSPs) play an important role in the interaction of *A. marginale* with host immune system, and the antibody responses are primarily directed towards MSP2 and MSP3. However, as the genes encoding these proteins can undergo continuous rearrangement the resulting antigenic variation means that immune responses to MSP2 and MSP3 do not confer life-long protection [3]. As a result, the high variability of these proteins makes them unsuitable for inclusion in vaccines targeting *A. marginale*.

Immunisation of cattle with *A. marginale* outer membrane (OM) fractions have induced complete protection against infection and disease [4,5,6]. Among the subdominant antigens identified in the OM are the type IV secretion system (T4SS) proteins. Several T4SS proteins induced type 1 immune responses against *A. marginale* infection; cluster of differentiation (CD)4^+^ T-cell responses, interferon gamma (IFN-γ) production and immunoglobulin G (IgG2) production, in cattle immunised with the OM proteins [7,8,9,10]. To identify *A. marginale* T4SS protein candidates for linked immune recognition that could be incorporated into a vaccine, Morse et al. [11] studied the specific interactions of VirB9-1 with VirB9-2 and VirB10, and demonstrated that VirB9 and VirB10 were highly immunogenic of the 11 *A. marginale* T4SS proteins examined for cattle with diverse major histocompatibility complex (MHC) class II haplotypes. They suggested that the T4SS VirB9 and VirB10 proteins may be desirable vaccine targets for the *Anaplasmatacae*. Moreover, Lopez and colleagues further demonstrated that three T4SS proteins (VirB9, VirB10 and conjugal transfer protein) were highly conserved with orthologous proteins in *Anaplasma phagocytophilum*, *Ehrlichia chaffeensis* and *Ehrlichia canis* [8]. Furthermore, the surface exposed components of VirB9-1 are highly conserved, making it an ideal candidate for inclusion in prototype vaccines against multiple *A. marginale* strains [12].

The over-expression of the OM proteins VirB9-1 and VirB10 has been challenging in bacterial expression systems. To date expression of VirB9-1 and VirB10 has been reported using the FLAG-tag (a polypeptide protein tag) or His-tag systems, resulting in insoluble products presumably due to their intrinsic properties as membrane proteins [11,13]. Recently, the methylotrophic *Pichia pastoris* has rapidly become a highly successful system for the expression of heterologous proteins and is considered faster, easier, and less expensive than insect or mammalian protein expression systems [14,15,16]. Proteins produced in *P. pastoris* are biologically active molecules, Wang and colleagues recently reported that yeast-expressed Epstein-Barr virus envelope glycoprotein gp350 retained strong immunogenicity in mice [17]. Ease of scale-up fermentation, lack of endotoxin production, and the capacity to facilitate secretion of the recombinant protein of interest into the yeast culture media make *P. pastoris* ideally suited for veterinary vaccine applications.

Due to the low immunogenicity of many purified recombinant proteins, they require the inclusion of adjuvants or carriers in subunit vaccine formulations to enhance antigen specific immune responses [18,19]. Recently, mesoporous silica nanoparticles (MSNs) have been successfully used as self-adjuvanting antigen carriers that stimulate strong, durable and specific immune responses to the major immunological determinant of bovine viral diarrhoea virus 1 [18,20,21,22]. Silica nanoparticles known as silica vesicles (SV) have been shown to be non-toxic, have excellent biocompatibility, and induce long-term humoral and cell mediated immune responses in mice [18,19,23]. The SV-100 nanoparticles have a diameter of 50 nm with a thin outer shell of 6 nm thickness, and a pore entrance size which can be modified within the range of 5.7 nm to 16 nm. Furthermore SV nanoparticles can be functionalised to fine tune protein adsorption [24]. A prior study has demonstrated the capacity of SV-100 nanoparticles to adsorb *Escherichia coli* expressed VirB9-1 and VirB9-2 and generate strong immune responses [25].

In this study we investigated the use of *P. pastoris* as an expression system to produce soluble recombinant VirB9-1 and VirB10 proteins. The immunogenicity of the expressed proteins in mice was tested following adsorption to self-adjuvanting SV-100 nanoparticles. Furthermore, a combined formulation of SV-100 adsorbed VirB9-1 and VirB10 proteins was tested to investigate the durable cell-mediated and antibody immune responses against *A. marginale*.

## 2. Results

### 2.1. Yeast Expression and Purification of Recombinant VirB9-1 and VirB10

**Figure 1:**
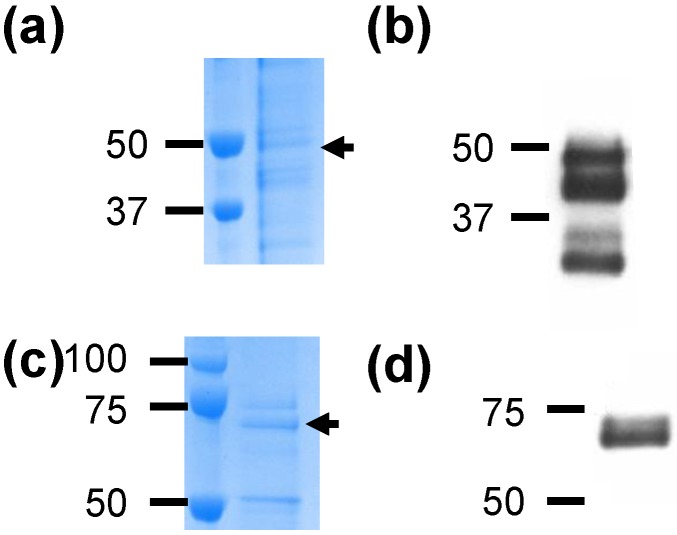
Expression and characterisation of VirB9-1 and VirB10. Purified proteins were loaded at 6 µg/well for Coomassie Blue stain (**a**,**c**), and 1.2 µg/well for Western blot (**b**,**d**); (**a**) VirB9-1 purified protein; (**b**) Western hybridisation of VirB9-1, probed with anti-VirB9-1 mAb (133/248.14.1.28); (**c**) VirB10 purified protein; (**d**) Western hybridisation of VirB10, probed with an anti-VirB10 mAb (138/481.3.9). Sizes shown in kDa. Full gel image of (**b**) and (**d**) is shown in Figure S2.

Recombinant VirB9-1 and VirB10 were successfully expressed using the *P. pastoris* system and purified from the culture media using metal affinity chromatography (Figure 1). The yields of the protein VirB9-1 (Figure 1a) and VirB10 (Figure 1c) were 42 µg/g cell pellet and 36 µg/g cell pellet, respectively. Some contaminating polypeptides were co-purified were observed as weak bands on the sodium dodecyl sulfate polyacrylamide gel electrophoresis (SDS-PAGE) analyses. Western blot analysis of purified VirB9-1, probed with an anti-VirB9-1 mAb (133/248.14.1.28) identified two of the purified proteins with estimated molecular weights of 24 and 48 kDa as VirB9-1 (Figure 1b). Western blot analysis of purified VirB10, probed with an anti-VirB10 (138/481.3.9) mAb identified a 60 kDa polypeptide as VirB10 (Figure 1d).

The corresponding polypeptides on Coomassie blue (R250) stained SDS-PAGE to the reactive polypeptides on the western analyses (Figure 1a,c) were excised for tandem mass spectrometric analysis (MS/MS analysis). The peptides consistent with VirB9-1 were identified in both the 24 kDa and 48 kDa bands, (uniprot:UPI0000497A73) [26], thus confirming the identity of these polypeptides as monomeric and dimeric VirB9-1. The MS/MS analysis identified VirB10 in a 60 kDa band (uniprot:UPI0000497DBF) [27] on the SDS-PAGE (Figure S1).

### 2.2. SV-100 Nanoparticle Adsorption Studies

**Figure 2:**
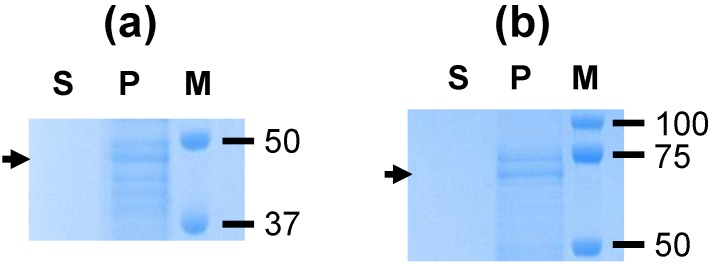
Sodium dodecyl sulfate polyacrylamide gel electrophoresis (SDS-PAGE) analyses demonstrating the high adsorption capacity of (**a**) VirB9-1 and (**b**) VirB10 to the SV-100 nanoparticles. The particle-protein slurry was mixed at 4 °C at 200 rpm overnight, and then assessed by electrophoresis. Supernatant (S) and pellet (P). SeeBlue^®^ Plus2 Protein Marker (M).

**Figure 3:**
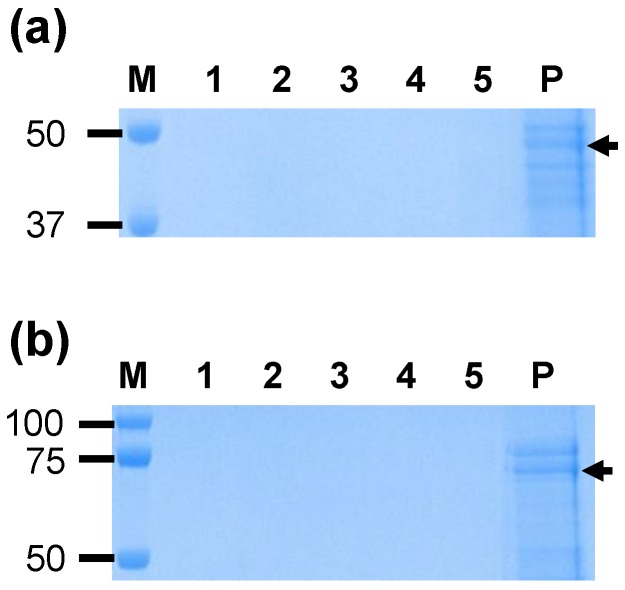
SDS-PAGE analysis of the desorption of (**a**) VirB9-1 and (**b**) VirB10 from SV-100 in phosphate-buffered saline (PBS) at different time points. Lane 1: Supernatant 5 min; lane 2: Supernatant 15 min; lane 3: Supernatant 30 min; lane 4: Supernatant 3 h; lane 5: Supernatant 24 h; lane 6: Pellet 24 h.

The silica vesicles SV-100 used for VirB protein adsorption Figure S3 have been well characterised previously [24,25]. SV-100 adsorption tests were conducted to determine the capabilities of SV-100 silica vesicles to bind to VirB9-1 and VirB10 proteins in phosphate-buffered saline (PBS) at 4 °C. Figure 2 illustrates that the SV-100 nanoparticles have an adsorption capacity of approximately 200 µg/mg SV-100 for both VirB9-1 (Figure 2a) and VirB10 (Figure 2b). No desorption of VirB9-1 or VirB10 from SV-100 was observed until after 24 h incubation at 37 °C in PBS (Figure 3). The stability study showed that VirB9-1 and VirB10 adsorbed onto SV-100 were more stable than VirB9-1 or VirB10 alone at both room temperature and 4 °C for a period of two months (Figure S4). TEM images of SV-100 before and after adsorption of VirB9-1 and VirB10 show no differences (Figure S3).

### 2.3. Evaluation of the Potential Cytotoxicity of the Immunisation Components

**Figure 4:**
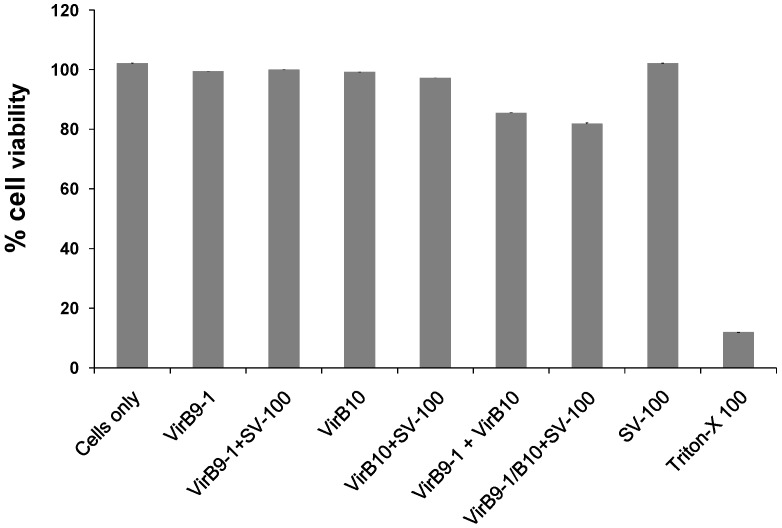
Cytotoxicity of the VirB9-1 and VirB10 in PBS, adsorbed to SV-100, combination of VirB9-1 and VirB10 with or without SV-100 were evaluated using the 3-(4,5-dimethylthiazol-2-yl)-2,5-diphenyltetrazolium bromide (MTT) assay at 10 μg/mL. Error bars indicate standard deviation.

The potential cytotoxicity of the yeast expressed proteins VirB9-1, VirB10, SV-100 and formulations were quantified using an 3-(4,5-dimethylthiazol-2-yl)-2,5-diphenyltetrazolium bromide (MTT) assay. A quantitative cytotoxicity analysis of the different treatments was conducted at 10 μg/mL concentrations in Madin-Darby bovine kidney (MDBK) cell lines. The MDBK cells were incubated with the treatment formulations for 24 h. The cells were treated with VirB9-1, VirB10, VirB9-1/VirB10, SV-100 alone, VirB9-1 + SV-100, VirB10 + SV-100, VirB9-1/VirB10 + SV-100 (Figure 4). The cell viability of all treatments groups was >80%. Overall the results suggested there was minimal cytotoxicity caused by the expressed proteins alone or when they were adsorbed to the SV-100 nanoparticles.

### 2.4. VirB9-1 and VirB10 Stimulate Specific Proliferation of Bovine T-Lymphocytes

**Figure 5:**
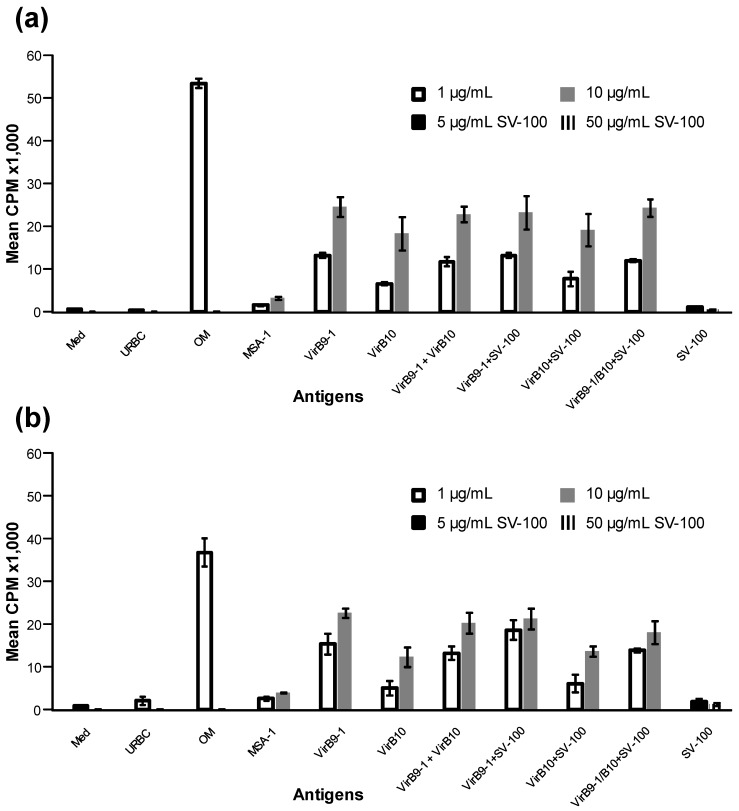
*A. marginale*-specific T-lymphocyte proliferation responses to VirB9-1 and/or VirB10 alone or adsorbed onto SV-100. Two-week T-cell lines established from *A. marginale* outer membrane (OM) -immunised calve numbers; 1 (**a**) and 2 (**b**) were tested for proliferation against 1 µg/mL and 10 µg/mL of VirB9-1 and/or VirB10 antigens alone or adsorbed onto SV-100 and; Med; media only (negative control), uninfected red blood cell membranes (URBC); Uninfected red blood cell membranes (negative control), OM; *A. marginale* outer membrane (positive control), merozoite surface antigen-1 (MSA-1); *Babesia bovis* merozoite surface antigen-1 (negative control protein), SV-100; SV-100 nanoparticles alone, tested at concentrations of 5 and 50 µg/mL. Error bars indicate standard deviation.

Bovine T-cell lines specific for *A. marginale* OM proteins derived from immunised calves 1 and 2 were used in proliferation assays to measure VirB9-1 and VirB10 protein-specific responses in vitro. In the case of cells from both animals, VirB9-1, VirB10 and VirB9-1/ViB10, alone and absorbed on SV-100, showed similar immune responses (Figure 5a,b). This shows that SV-100 can be successfully used in a mixed antigen formulation with no reduction in immune response compared to individual formulations. There were no detectable responses to 5 and 50 µg/mL of SV-100.

### 2.5. Mouse Antibody Responses to VirB9-1 and VirB10 Formulated with SV-100

**Figure 6:**
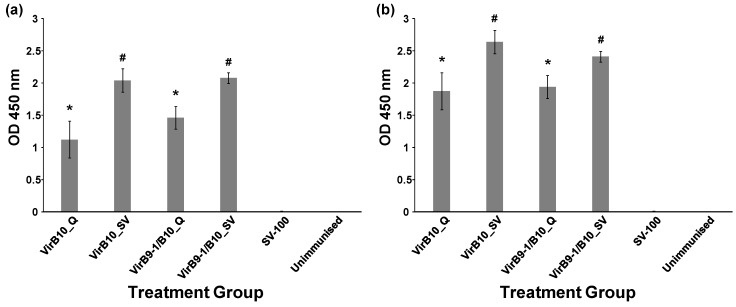
The SV-100 silica vesicles act as a self-adjuvanting antigen carrier and induce potent and sustained immune responses to *A. marginale* antigens in mice. Sera were collected 2 weeks after the final immunisation. (**a**) VirB9-1 specific antibody total production and immunoglobulin G (IgG) level (1:1600 dilution) in C57BL/6J mice for different vaccine formulation groups; (**b**) VirB10-specific antibody total IgG level (1:1600 dilution) in C57BL/6J mice for different vaccine formulation groups. Error bars indicate standard error. Groups marked with **#** show significant differences (*p* < 0.05) from the groups marked with *. Table S1, shows the ELISA data from the full dilution range of 1:100–1:6400.

The antibody responses stimulated in mice following immunisation with the SV-100 and QuilA formulations (Table 1), were determined by Enzyme-Linked ImmunoSorbent Assay (ELISA). The ELISA results from the terminal bleeds for VirB9-1 and VirB10 show immunogenic activity, and eliciting strong antibody responses (Figure 6). The specific anti-VirB9-1 IgG responses (Figure 6a) generated by the group injected with VirB9-1_SV were stronger than VirB9-1_Q (*p* = 0.035). Similarly, stronger immune responses were detected in VirB9-1/B10_SV, than the mixed VirB9-1B10_Q formulation group (*p* = 0.015). Furthermore, higher anti-VirB10 IgG responses (Figure 6b) were also observed in VirB10_SV (*p* = 0.035) and VirB9-1/B10_SV (*p* = 0.0197) compared to the analogous QuilA groups.

The negative control groups receiving either SV-100 or the unimmunised group showed no detectable VirB9-1-specific or VirB10-specific antibody responses. Overall, the levels of anti-VirB9-1/VirB10-specific IgGs induced by SV-100 adsorbed proteins were significantly higher than that induced by QuilA.

### 2.6. Cell Mediated Immune Responses to VirB9-1 and VirB10 Formulated with SV-100

Fluorescence activated cell sorting (FACS) following intracellular staining for selected cytokines was conducted to study the mouse spleen cell immune response to VirB9-1 and ViB10 formulated with SV-100 nanoparticles and QuilA.

**Figure 7:**
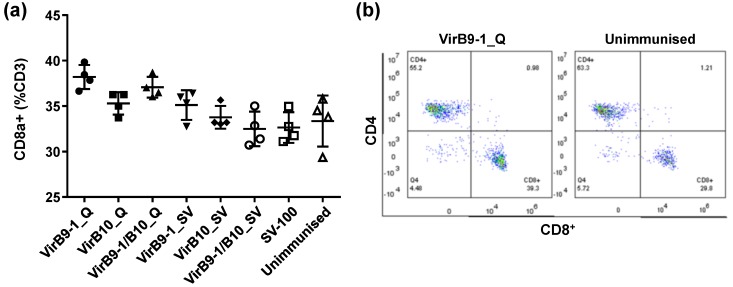
Fluorescent activated cell sorting (FACS) of spleen cells from immunised mice showing CD8^+^ population of CD3 in treatment groups. Gating strategies for the spleen inflammatory infiltrate. Cells were first gated on size SSC-A: Side Scatter of cells-Area vs. FSC-A: Forward Scatter of cells-Area, and viability DRAQ5 (The Thermo Scientific DRAQ5™ Fluorescent Probe) expression, not shown, then leukocytes (CD45^+^ cells) were sub-gated for CD4^+^ and CD8^+^ population. (**a**) CD8^+^ population of CD3 in all treatment groups; (**b**) Gating strategies of VirB9-1_Q and unimmunised groups.

The FACS analysis on the CD8^+^ T-cell population of unstimulated mouse spleen cells, showed no significant differences between other treatment groups and the control group except for the VirB9-1_Q group (Figure 7a) This difference was significantly higher than that in control group (Figure 7b, 39.3% in VirB9-1_Q verses 29.8% in the unimmunised group, *p* = 0.021).

**Figure 8:**
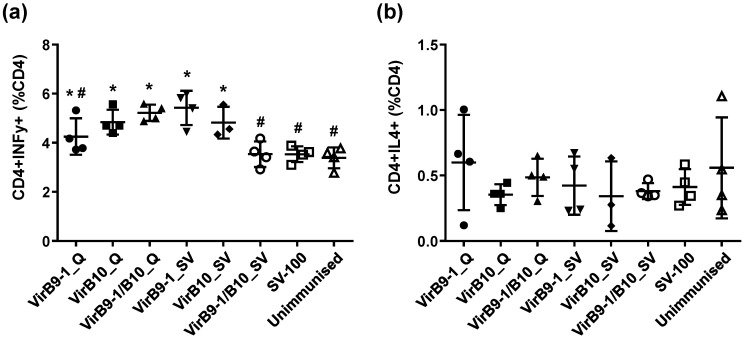
FACS analysis of mice (*n* = 4) spleen T lymphocyte sub-populations after re-stimulation with VirB10 protein for 3 h. (**a**) Cell populations with Th1 phenotype in treatment groups after re-stimulation. Groups marked with **#** show significant differences (*p* < 0.05) from the groups marked with *. VirB9-1_Q shows no significant differences with any other group (*#); (**b**) Cell populations with Th2 phenotype in treatment groups after re-stimulation, no significant differences are seen (*p* < 0.05).

After re-stimulating with VirB10, the Type 1 helper T cells (Th1) cytokine profile from VirB10_Q (*p* = 0.0047), VirB9-1/VirB10_Q (*p* = 0.0005) treatment groups were all significantly higher compared to the unimmunised control (Figure 8a). VirB9-1_SV (*p* = 0.0026), and VirB10_SV (*p* = 0.017) also showed significant responses compared to the SV-100 control, however the combined group of VirB9-1/B10_Q did not show any significant response (Figure 8a). Interestingly, VirB10 re-stimulated Type 2 helper T cells (Th2) immune response did not show any significant difference between the groups (Figure 8b). FACS analysis of VirB9-1 re-stimulated cells were inconsistent.

## 3. Discussion

Although cattle produce antibodies against the major surface proteins MSP2 and MSP3 following infection with *A. marginale*, the immune responses are not effective in protecting against new infections or reinfections as MSP2 and MSP3 are highly variable. As a result, efforts have been made to identify and develop alternative antigens, such as VirB9-1 and VirB10, two immunologically subdominant proteins from *A. marginale* outer membranes for use in immunisation and protection studies [11]. However, these efforts have been hindered by difficulties in producing soluble VirB9-1 and VirB10 proteins for use in prototype vaccine formulations. Therefore, it is desirable to express soluble VirB9-1 and VirB10 proteins. VirB9-1 and VirB10 have been previously expressed in bacteria using FLAG-tag or His-tag technologies, however both yielded insoluble proteins likely due to their intrinsic properties as outer membrane proteins [11,13]. This issue has been addressed in the current study, by producing soluble VirB9-1 and VirB10 using the yeast *P. pastoris* expression system. *P. pastoris* has rapidly become a highly successful system for the expression of heterologous proteins [16]. Proteins produced by *P. pastoris* are more likely to be processed, correctly folded, and post-translationally modified. VirB9-1 and VirB10 expressed in our laboratory have been confirmed with highly specific mAb by western hybridisation analysis as well as MS/MS analysis. As other *A. marginale* proteins were not identified, this is consistent with the formation of homodimers by VirB9-1 and VirB10 (Figure 1) as previously reported [11]. The protein bands seen in Figure 1b likely represent degradation products of the individual proteins resulting in bands smaller than the monomers and dimers, as previously observed for these proteins [8].

It is unusual to increase the complexity of the expression system compared to the original source of the antigen as we have done in this study by expressing a prokaryotic antigen in a eukaryotic host. However, in the case of VirB9-1 and VirB10, this strategy has enabled the production of soluble proteins that facilitated the formulations of the antigens with SV-100 nanoparticles for immunisation studies. Furthermore, expression of VirB10 protein in *E. coli* is difficult, with extremely low yields. Gandhi and colleagues demonstrated a two-fold increase in the yield of *Geobacillus stearothermophilus* derived SR74 recombinant β-Amylase using the *P. pastoris* expression system compared to an *E. coli* expression system [28].

Importantly the yeast-expressed antigens were able to stimulate T-cell lineages derived from calves immunised with the OM proteins of *A. marginale*, thus demonstrating the immunological integrity of the recombinant VirB9-1 and VirB10. These results suggest that immunisation of cattle with VirB9-1 and VirB10 formulated with SV-100 would induce specific immune responses to these *A. marginale* antigens. Although beyond the scope of the current study, it would be of interest to determine if these SV-100 formulations can stimulate primary responses in cattle and also protect them from *A. marginale* challenge.

The efficient adsorption of protein to SV-100 nanoparticles is likely to enhance the uptake of antigen by antigen presenting cells. The immunogenicities of yeast expressed VirB9-1 and 10 were studied using SV-100 nanoparticles as adjuvants compared to the commercial adjuvant QuilA. The adsorption capacity of proteins to the silica vesicles is highly dependent on the physicochemical characteristics of both proteins and silica vesicles. Proteins with hydrodynamic diameter that is smaller than the pore entrance diameter can easily enter the mesopores, while the ones with larger diameter adsorb on the outer surface of the nanoparticles [29]. The specially designed novel SV-100 (50 nm diameter) silica vesicles with high protein adsorption capacities [24] have been shown to be a potential self-adjuvant with high levels of both humoral and cell-mediated immune responses in mice with no detrimental toxicity effects [18]. The SV-100 which has an entrance size of ~6 nm has been shown to be efficiently up-taken by dendritic cells, and its spherical shape similar to a virus also enhances higher antibody response compared to other shapes of similar size [19]. In this study, both VirB9-1 and VirB10 were fully absorbed within SV-100 silica vesicles at ratio of 200 µg antigen per mg of SV-100. The absorbed protein release experiment of ViB9-1/VirB10 adsorbed SV-100 at 37 °C in PBS buffer showed that VirB9-1/VirB10 once adsorbed to SV-100 did not dissociate immediately, a similar result to previously reported study [18]. The efficient adsorption of VirB9-1/VirB10 to SV-100 and slow dissolution of protein adsorbed SV-100 is likely to enhance the uptake of antigen by antigen presenting cells.

Ideally, adjuvants have a multipurpose role in vaccine formulation, to enhance the immunogenicity of highly purified or recombinant antigens, reduce the amount of antigen required and/or reduce the number of immunisations needed for protective immunity. The capacity of SVs to initiate both humoral and cell mediated immune responses in the absence of a conventional adjuvant has previously been reported when used to deliver the bovine viral diarrhoea virus 1 protein E2 in a mouse model [23]. Zhao and colleagues recently demonstrated that SV-100 as the carrier and adjuvant to deliver *E. coli*-expressed VirB9-1 and VirB9-2 induced high levels of IgG and cell-mediated immune responses that are comparable to those induced by QuilA [25].

The FACS results of mouse spleen cell surface markers showed that the CD8^+^ T-cell population in QuilA treatment group, was much higher than that in the unimmunised control group (Figure 7). This is consistent with the previous studies showing that QuilA is a strong adjuvant inducing responses to T-dependent as well as T-independent antigens; it can also induce strong cytotoxic CD8^+^ T-lymphocyte responses and potentiate the response to mucosal antigens [30,31]. From previous studies, CD8^+^ effector/memory T-cells and CD4^+^ memory-phenotype cells have different life spans in mouse models (14 to 60 days of CD4^+^ or 20 to 91 days of CD8^+^ memory cell) [32,33,34,35]. Thus the continual stimulation observed in this study could be triggered via the depot effect that could sustain sufficient and vigorous CD8^+^ and CD4^+^ effector/memory T-cells throughout 5 weeks after second inoculation, promoting efficient cellular responses. It is well known that QuilA-adjuvanted vaccines induce a mixed Th1/Th2 response characterised by increased levels of both IgG1 and IgG2a [36]. Morse and colleagues demonstrated the special interactions of VirB9-1 and VirB10, and revealed that VirB9-1 and VirB10 are interacting protein partners [11]. The cattle T-lymphocyte proliferation assay results further confirmed that yeast-expressed VirB9-1 and VirB10 are highly active in eliciting a T-cell-mediated recall response.

CD8^+^ T lymphocytes are also excellent sources of IFN-γ [37]. Research on *A. marginale* in cattle immunised with protective vaccines is consistent in identifying CD4^+^ T-cells, IFN-γ secretion, and activation of specific IgG isotypes for neutralisation and opsonisation as determinants of protective immunity [5]. Indeed, a strong cellular response characterised by IFN-γ and IgG2 production is important for protective immunity of anaplasmosis [38]. This study demonstrated that IFN-γ was expressed in response to VirB9-1 and VirB10, indicating that VirB proteins produced in the yeast expression system could be potent antigens for cattle immunisation.

## 4. Materials and Methods

### 4.1. Cloning of VirB9-1 or VirB10 into pPICZαA Expression Vector

The open reading frame (ORF) encoding VirB9-1 was release by restriction endonuclease digestion of pMA-T_VirB9-1 (Life Technologies, Carlsbad, CA, USA) with Eco*RI* and Xba*I*. The resultant VirB9-1 ORF was ligated into similarly digested pPICZαA vector (Life Technologies). The ORF encoding VirB10 was synthesised and supplied cloned into pPICZαA as pPICZaA_VirB10 (Life Technologies). The ligation products were confirmed and subsequently transformed into *E. coli* strain One Shot Top 10 (Invitrogen, Waltham, MA, USA). Positive clones were confirmed by sequencing (AGRF, Brisbane, Australia) and then purified and linearised plasmid was electroporated into *P. pastoris* strains X-33, KM71H, and GS115 strains and subsequent plating on media containing Zeocin. Transformed clones were identified using PCR analysis of *P. pastoris* integrants (5′ AOX1 primer and 3′ AOX1 primers) was performed following the manufacturers. The capacity of PCR positive clones for each strain to express VirB9-1 were confirmed with SDS-PAGE gels by both Coomassie R250 staining and western blot using small-scale expression. The highest expressing strain X-33 was selected for large-scale expression.

### 4.2. Large-Scale Expression and Purification of VirB9-1 and VirB10 Proteins

An overnight culture of a single X-33_VirB9-1 or XX-33_VirB10 colony in 2 mL of Buffered glycerol-complex medium (BMGY) was used to inoculate 25 mL cultures of BMGY (Life Technologies).These cultures were grown at 28 °C in a shaking incubator (260 rpm) to an optical density at 600 nm (OD_600_) of 2–6. The yeast cell pellets were collected by centrifugation at 2000× *g*, at 20 °C for 5 min. The supernatant was decanted and discarded. The cell pellet was re-suspended in Buffered Methanol-complex Medium (BMMY) containing 0.5% methanol to an OD_600_ of 1.0 to induce expression. Methanol (100%) was added to a final concentration of 0.5% methanol every 24 h to maintain induction. The supernatants and cell pellets were collected and stored at −80 °C for protein purification after 72 h of methanol induction. The cell pellets were homogenised by Polytron^®^ homogeniser (Kinematica AG, Luzern, Switzerland) in lysis buffer (100 mM NaH_2_PO_4_, 10 mM Tris-Cl, 8 M Urea, pH 8.0). The lysates were incubated for 30 min with gentle shaking. The resultant solution was centrifuged at 15,000× *g* at 4 °C for 30 min. The supernatants were stored at −80 °C until required. Purification of 6-His-tagged proteins were performed with Ni-NTA Agarose (QIAGEN, Hilden, Germany) following the manufacturer’s instructions.

### 4.3. SDS-PAGE Electrophoresis

SDS-PAGE analysis was performed using Invitrogen’s XCell SureLock^®^ Mini-Cell precast system with NuPAGE 10% BIS-Tris gels according to manufacturer’s instructions. Purified proteins were loaded at 6 µg per well. Size estimations were determined against SeeBlue^®^ Plus2 (Invitrogen) or Precision Plus Protein Kaleidoscope Standards (BioRad, Hercules, CA, USA) pre-stained standards. The resolved proteins were visualised by staining in 50% methanol, 10% acetic acid, 0.25% Coomassie Blue R250 for 30 min, followed by de-staining in 30% methanol, 10% acetic acid for 20 min three times.

### 4.4. Western Hybridisation Analysis

Purified proteins were loaded at 1.2 µg per well. Following SDS-PAGE electrophoresis the resolved polypeptides were transferred to Hybond C nitrocellulose membrane (GE Healthcare, Buckinghamshire, UK) using Invitrogen XCell II™ Blot Module Kit according to manufacturer’s instructions. The membranes were probed with the monoclonal antibodies 133/248.14.1.28 or 138/481.3.9 which specifically bind VirB9-1 and VirB10 respectively at dilutions of 1:3,000. Anti-mouse immunoglobulin G HRP conjugate (Chemicon, Millipore, Billerica, MA, USA) was used at 1:80,000. Detection was carried out using an ECL detection kit (Thermo, Rockford, IL, USA).

### 4.5. Tandem Mass Spectrometric Analysis of VirB9-1 and VirB10

Polypeptide bands corresponding to 24 and 48 kDa for VirB9-1 and 60 kDa for VirB10 were excised from the Coomassie stained gels, destained (50% methanol, 5% acetic acid), dehydrated with 100% acetonitrile, reduced with 10 mM DTT, quenched with 50 mM iodoacetemide, and digested with 20 ng/μL of trypsin (Promega, Fitchburg, WI, USA). The peptides were subjected to tandem mass spectrometric (MS/MS) fractionation on a high performance liquid chromatography (HPLC) coupled quadruple-time of flight (Q-TOF) MS instrument located at The University of Queensland, Australian Institute for Bioengineering and Nanotechnology. Fragment ion lists and the identified peptide sequences were searched against the MASCOT database that contained *A. marginale*, *E. coli*, or all National Center for Biotechnology Information (NCBI) entries. Identification of the protein was based upon the score given, probability, and by mass. One missed trypsin cleavage, fixed carbamidomethyl modifications, and variable oxidation was allowed during the search. A probability of 95% or greater showed that the peptide match was not a random occurrence, and the individual ion score is reported as −10log_10_P, where P is the probability. An ion score greater than 19 was considered to represent significant identity.

### 4.6. SV-100 and VirB9-1 and VirB10 Absorption Assay

SV-100 nanoparticles were produced as previously described [24,25]. Adsorption reactions used 2 mg of SV-100 with 400 μg of VirB9-1 or VirB10 in sterile PBS in a 2 mL final volume. This particle-protein slurry was placed on a shaker at 4 °C at 200 rpm. After 24 h a sample of particle-protein slurry (50 μL) was removed and centrifuged at 13,000× *g* for 1 min. The amount of unbound protein was assessed by electrophoresis of the supernatants and the nanoparticles on SDS-PAGE gels. Protein assay was conducted on the adsorbed supernatant, using the BioRad DC kit, to quantify the amount of the unbound protein to estimate the amount of protein bound to the nanoparticles.

### 4.7. Desorption Studies

The VirB9-1 or VirB10 bound SV-100 vesicle pellets were re-suspended in 1 mL of PBS. The re-suspended samples were left on the shaker at 37 °C for 24 h at 200 rpm. Aliquots (50 μL) were taken at 5 min, 15 min, 30 min, 3 h and 24 h. The protein released from the SV-100 was assessed by electrophoresis of the supernatants and the nanoparticles by SDS-PAGE analysis.

### 4.8. Stability Assay

The proteins stability assay was performed from VirB9-1 or VirB10 bound SV-100 vesicle pellets which were stored at room temperature or at 4 °C for different time periods. Same amount of protein from each testing sample was loaded and analysed by SDS-PAGE.

### 4.9. Cell Viability Assay

The cytotoxicity of the yeast expressed proteins in PBS, VirB9-1, VirB10, VirB9-1/SV-100, VirB10/SV-100 was quantified using the colorimetric assay (MTT based). In a 96 wells flat bottom cell culture plate, the MDBK cells were seeded at 5 × 10^4^ cells per well in 100 μL of Earle’s Minimum Essential Media with 5% FBS, and incubated overnight in at 37 °C, 5% CO_2_. The next day, the expressed proteins or protein/SV-100 complexes were added to the cells at concentrations of 10 μg/mL. Triton X-100 at a concentration of 100 μg/mL was used a positive control for cell death, and the plate was incubated overnight at 37 °C, 5% CO_2_. Next day, 10 μL of MTT (0.5 mg/mL final concentration) was added to each well and the plate was further incubated for 4 h at 37 °C, 5% CO_2_. After 4 h, 65 μL of the media was removed from the wells and 50 μL of dimethyl sulfoxide (DMSO) was added to the wells, the plate was further incubated for 10 min at room temperature (RT) and then the optical density at 540 nm of each well was recorded using a BioTek microplate reader (BioTek, Winooski, USA).

### 4.10. Bovine A. marginale-Specific T-Lymphocyte Proliferation Assays

Holstein cattle calves 1 and 2 were immunised with *A. marginale* St. Maries strain outer membranes (OM) as described [11] with the exception that calves 1 and 2 were immunised four times at three week intervals with 60 µg OM in QuilA [11]. Two-week T-cell lines were obtained by stimulating 4 × 10^6^ peripheral blood mononuclear cells (PBMC) in complete RPMI-1640 medium with 5 µg/mL OM for one week in 1.5 mL volumes in 24-well plates. Cells were harvested and washed in complete RPMI-1640 medium, and viable cells obtained after Ficoll-Hypaque purification, if needed, were re-cultured at 7.5 × 10^5^ cells per well with 2 × 10^6^ irradiated autologous PBMC in 1.5 mL complete RPMI-1640 without antigen for one week (resting). Cells were harvested and viable cells were cryopreserved in liquid nitrogen in a mixture of 10% DMSO in foetal bovine serum for use in proliferation assays.

Proliferation assays were carried out in replicate wells of round-bottomed 96-well plates for 3 days using the cryopreserved two-week T-lymphocyte lines from calves 1 and 2 as described previously [11]. The T-cells (2 × 10^4^ cells) were cultured in replicate wells in a total volume of 100 µL of complete RPMI-1640 medium containing antigen and 2 × 10^5^ irradiated autologous PBMC as a source of antigen presenting cells. The VirB9-1 and VirB10 proteins were used at a final concentration of 1 and 10 μg/mL in complete RPMI-1640 medium or a mixture of the two at a final protein concentration of 2 and 20 µg/mL. Positive controls include *A. marginale* OM used at 1 µg/mL and negative controls included uninfected red blood cell membranes (URBC), and *B. bovis* merozoite surface antigen-1 (MSA-1) used at 1 and 10 μg/mL. SV-100 adsorbed with VirB9-1, VirB10, or a mixture of the two were tested at final concentrations of VirB9 proteins of 1 and 10 µg/mL or 2 and 20 µg/mL for the mixture. The SV-100 nanoparticles were tested at 2.5, 5.0, 7.5, 10, 25, 50, 75 and 100 µg/mL in the absence of antigen as negative controls. T-cell proliferation was quantified by incorporation of 0.25 μCi per well ^3^H-thymidine (Dupont, New England Nuclear, Boston, MA, USA) during the last 6 h of culture. The radiolabelled DNA was harvested (Tomtec Cell harvester, Ontario, Canada) on glass filters and the emitted β-particles were counted with liquid scintillation. Results are presented as the mean counts per minute (cpm) +/− 1 SD. The cpm of the different antigens stimulations were compared to the cpm for the matching concentration of MSA-1 with Boneferroni-Holm (one-way ANOVA with posthoc test). Statistically significant T-cell stimulation by a treatment was set at a *p*-value < 0.05.

### 4.11. Immunisation Studies Conducted in Mice

C57BL/6J mice were purchased from and housed in the Biological Resource Facility, The University of Queensland, Brisbane, Australia under specific pathogen-free conditions. Eight week old female mice were housed in HEPA-filtered cages with 4 animals per group (8 groups, Table 1) in an environmentally controlled area with a cycle of 12 h of light and 12 h of darkness. Food and water were given *ad libitum*. All procedures were approved by The University of Queensland Ethics Committee (Approval number: 239/14). Animals were closely monitored throughout the study. All the animals remained in good health for the duration of the study with no visible deleterious health effects.

Pre-immunisation blood samples were collected by retro-orbital bleeds using heparin coated haematocrit tubes (Hirschmann Laborgeräte, Heilbronn, Germany). Pre-immunisation blood samples collected prior to the first immunisation were referred to as the pre-immune (PI) samples. Table 1 shows the different treatment groups in the study. QuilA (Superfos Biosector, Vedback, Denmark) was re-suspended at 2 mg/mL in sterile injectable water (Pfizer, Brooklyn, New York, NY, USA). The negative control group received injections of SV-100 (250 μg) alone. Dose volumes of 100 µL (in 0.9% saline, Pfizer, Sydney, Australia) were administered by subcutaneous injection at the tail base using a sterile 27 gauge needle (Terumo, Tokyo, Japan). Two injections were administered at 2 week intervals to all the treatment groups except for the unimmunised group and mice were sacrificed 35 days after the second immunisation. The antibody responses were studied using the Enzyme-Linked ImmunoSorbent Assay (ELISA) assay and cell-mediated responses were analysed using fluorescence activated cell sorting (FACS) following staining for intracellular selected cytokines.

### 4.12. ELISA Assay

An ELISA for the detection of VirB-specific antibodies were performed by coating microtitre plates (96 well, Nunc, Maxisorb, Roskilde, Denmark) with VirB9-1 or VirB10 antigen solution (2 ng/µL, 50 µL per well) in PBS overnight at 4 °C. The coating solution was removed and the plates were washed once with PBS-T (PBS containing 0.1% Tween-20) and blocked with Bovine Serum Albumin (BSA, 5%, Sigma-Aldrich, St. Louis, MO, USA) and skim milk (5%, Fonterra, Auckland, New Zealand) in PBS (200 µL) for 1 h with gentle shaking at RT. Plates were washed three times with PBS-T.

Mouse sera samples, in duplicate, were diluted from 1:100 to 1:6, 400 in PBS (50 µL) and each dilution was added to the wells of the blocked plates followed by incubation for 2 h at RT. To detect mouse antibodies Horseradish Peroxidase (HRP) conjugated polyclonal sheep anti-mouse IgG antibodies (Chemicon Australia, Melbourne, VIC, Australia) diluted in PBS to 1:40,000 were added to each well and incubated for 1 h at RT with gentle shaking. Plates were washed three times in PBS-T. TMB substrate (100 µL, Sigma-Aldrich, Castle Hill NSW Australia) was added to each well and incubated for 10 min at RT; HCl (1 N, 100 µL) was added to wells to stop the chromogenic reaction. The optical density at 450 nm of each well was recorded using a BioTek microplate reader (Bio Tek, Winooski, VT, USA).

### 4.13. Intracellular Staining for Selected Cytokines and FACS

Single cell suspensions of spleen were prepared by passing tissues through a 70 μm nylon cell strainer (Becton Dickinson, Franklin Lake, NJ, USA) into PBS containing 0.1% BSA (Sigma-Aldrich, St. Louis, MO, USA) and 0.1% sodium azide (NaN_3_; Sigma-Aldrich) (PBA). Spleen cell suspensions were incubated in lysis buffer (155 mM NH_4_Cl, 10 mM KHCO_3_, 0.1 mM EDTA; all from Sigma-Aldrich, St. Louis, MO, USA) for 10 min at 4 °C to lyse red blood cells. Cells were washed in PBA and distributed into clear, U-bottom 96 well plates (1.5–2.0 × 10^6^ cells/well) before centrifugation at 800 × *g* for 5 min at 4 °C. Supernatants were removed and cells pre-incubated with blocking buffer (5% normal rat serum and anti-CD16/32 (TruStainFcX, 1/50; BioLegend, San Diego, CA, USA) in PBA) for 15 min prior to surface staining with fluorophore-conjugated monoclonal antibodies (MAb) (α-CD45 PE/Cy7; α-CD4 PE; α-CD3 Alex 488; α-CD8a Alex 647. All antibodies were from BioLegend, San Diego, CA, USA) or the appropriate isotype controls at 4 °C for 30 min. For Th1, Th2 staining, cells were first stimulated for 3 h (37 °C) with VirB9-1 or VirB10 before the presence of Brefeldin A (5 μg/mL; BioLegend, San Diego, CA, USA), and then incubated overnight at 37 °C. After surface staining (α-CD45 PE-cy7; α-CD4 PE, all from BD Bioscience, San Diego, CA, USA), cells were fixed with Fixation Buffer and permeabilised with Permeabilisation/Wash Buffer (BioLegend) before incubation for 40 min at room temperature with fluorophore-conjugated antibodies to IL-4, or IFN-γ (α-IL-4 FITC; α-IFN-r Alex 647, all from BD). After antibody staining, cells were washed twice in PBA, then re-suspended in 100 μL PBA. All cells were analysed using an Accuri C6 flow cytometer (BD Biosciences, San Jose, CA, USA) and data analysed using FlowJo software package (Tree Star Inc., Ashland, OR, USA).

### 4.14. Statistical Analysis

Data were analysed by one-way ANOVA with Bonferroni-Holm post-tests aided by Daniel’s XL Toolbox for Excel, version 7.0.1, by Daniel Kraus, Würzburg, Germany (www.xltoolbox.net); *p*-values < 0.05 were considered statistically significant.

## 5. Conclusions

Soluble endotoxin free, and immunogenic VirB9-1 and VirB10 were successfully produced using the yeast *P. pastoris* expression system. The purified antigens, when formulated with Quil adjuvant, induced strong humoral and cell-mediated immune responses. The study also show that the yeast expressed antigens can be successfully delivered after adsorption onto SV-100 nanoparticles, as single and mixed formulations. The SV-100 adsorbed antigens elicited strong humoral and cell mediated (T-cell proliferation) immune responses. This further demonstrates the ability of SV nanoparticles to act as effective antigen carriers and adjuvants and the possibility of delivering multiple antigens using SV-100 formulations for next generation vaccines.

## Figures and Tables

**Table 1:** Immunisation groups in mice trial. All doses were administered at the tail base.

Group	Designation	Prototype Vaccine/Injection Dose
1	VirB9-1_Q	VirB9-1 (50 µg) + QuilA (10 µg)
2	VirB10_Q	VirB10 (50 µg) + QuilA (10 µg)
3	VirB9-1/B10_Q	VirB10 (50 µg) + VirB9-1 (50 µg) + QuilA (10 µg)
4	VirB9-1_SV	VirB9-1 (50 µg) + SV-100 (250 µg)
5	VirB10_SV	VirB10 (50 µg) + SV-100 (250 µg)
6	VirB9-1/B10_SV	VirB10 (50 µg) + VirB9-1 (50 µg) + SV-100 (250 µg)
7	SV-100	SV-100 (250 µg) alone
8	Unimmunised	Unimmunised control

## Supplementary Materials

The following are available online at http://www.mdpi.com/2079-4991/6/11/201/s1.

## Abbreviations

The following abbreviations are used in this manuscript:

OM	Outer membrane
RT	Room Temperature
SV	Silica vesicles
T4SS	Type IV secretion system
