# Supplementary material for: Nanoparticle-Based Delivery of Anaplasma marginale Membrane Proteins; VirB9-1 and VirB10 Produced in the Pichia pastoris Expression System
Source: Nanomaterials (Basel). 2016 Nov 5;6(11):201. doi: 10.3390/nano6110201 (PMC5245741; doi:10.3390/nano6110201)
Supplement: Supplementary file 1 [file nanomaterials-06-00201-s001.pdf]

Supplementary Materials: Nanoparticle Based Delivery of *Anaplasma marginale* Membrane Proteins; VirB9-1 and VirB10 Produced in the *Pichia pastoris* Expression System

Bing Zhang, Antonio S. Cavallaro, Karishma T. Mody, Jun Zhang, James R. Deringer, Wendy C. Brown, Timothy J. Mahony, Chengzhong Yu and Neena Mitter

MS data file : 20140703_Chang_gel_sequence2.mgf
Database : LudwigNR (25,913,601 sequences; 9,279,221,698 residues)
Timestamp : 6 Jul 2014 at 23:31:27 GMT

Not what you expected? Try the select summary.

▸Search parameters
▸Score distribution
▸Legend

Protein Family Summary

Significance threshold p< 0.05 Max. number of families AUTO
Ions score or expect cut-off 0 Dendrograms cut at 0
Preferred taxonomy All entries

Protein families 1–10 (out of 46)

10 per page 1 2 3 4 5 Next

▾1 Q5PBS8 2456 tr|Q5PBS8|trbG Conjugal transfer protein trbG n...

		Score	Mass	Matches	Sequences	emPAI
1.1	Q5PBS8	2456	29907	191 (106)	16 (12)	7.27

tr|Q5PBS8|trbG Conjugal transfer protein trbG n=1 Tax_Id=234826 [Anaplasma marginale (strain St. Maries)]

▾191 peptide matches (25 non-duplicate, 166 duplicate)

Query	Dupes	Observed	Mr(expt)	Mr(calc)	ppm	M	Score	Expect	Rank	U	Peptide
80	▸7	375.6986	749.3827	749.4072	-32.7	0	47	0.39	▸1	U	R.LAYEVR.F
1081	▸39	445.7489	889.4833	889.5022	-21.2	0	69	0.0057	▸1	U	R.VSPAFTLR.Y
1383	▸2	485.2481	968.4816	968.4927	-11.5	0	29	0.15	▸1	U	R.SIAADDHIK.I
2758	▸40	574.8166	1147.6186	1147.6237	-4.43	0	65	0.0015	▸1	U	R.IVGDYVVVER.V
3790	▸15	446.9051	1337.6936	1337.7204	-20.1	0	68	0.00092	▸1	U	K.IINFNPQSIHR.Y
3803	▸7	669.8717	1337.7288	1337.7204	6.27	0	95	1.1e-05	▸1	U	K.IINFNPQSIHR.Y
3941	▸21	681.8095	1361.6044	1361.5922	9.02	0	103	1.6e-07	▸1	U	R.YGSSTACVFNEK.L
5429	▸1	598.9535	1793.8388	1793.8407	-1.05	1	48	0.0014	▸1	U	R.YGSSTACVFNEKLYR.T
5998	▸6	704.3387	2109.9944	2109.9643	14.2	0	79	1.6e-06	▸1	U	R.VYYFELHAEEASGLDDPR.L
6007	▸1	1056.0257	2110.0368	2109.9643	34.3	0	63	0.00054	▸1	U	R.VYYFELHAEEASGLDDPR.L
6079	▸2	551.0539	2200.1864	2200.1852	0.56	0	60	0.0024	▸1	U	R.LFIKPVGDNADTNVTIITNR.R
6087	▸8	734.4151	2200.2234	2200.1852	17.3	0	99	7.3e-07	▸1	U	R.LFIKPVGDNADTNVTIITNR.R
6091		1101.1436	2200.2727	2200.1852	39.8	0	82	5.1e-06	▸1	U	R.LFIKPVGDNADTNVTIITNR.R
6156		555.2863	2217.1161	2217.1178	-0.76	0	20	0.54	▸1	U	K.GLNFDYSVSHTAGSANIVPIR.V
6162	▸7	740.0589	2217.1547	2217.1178	16.6	0	138	9.2e-12	▸1	U	K.GLNFDYSVSHTAGSANIVPIR.V
6167		1109.6090	2217.2035	2217.1178	38.6	0	73	6.6e-06	▸1	U	K.GLNFDYSVSHTAGSANIVPIR.V
6321	▸4	567.5239	2266.0664	2266.0654	0.42	1	79	1.1e-05	▸1	U	R.RVYYFELHAEEASGLDDPR.L
6328		756.3759	2266.1060	2266.0654	17.9	1	59	0.0011	▸1	U	R.RVYYFELHAEEASGLDDPR.L
6379		573.0587	2288.2057	2288.2025	1.40	1	18	5.6	▸8	U	R.SIAADDHIKIINFNPQSIHR.Y
6504	▸1	587.3126	2345.2212	2345.2128	3.58	1	90	2.1e-06	▸1	U	K.KGLNFDYSVSHTAGSANIVPIR.V
6505		782.7615	2345.2626	2345.2128	21.2	1	69	7.3e-05	▸1	U	K.KGLNFDYSVSHTAGSANIVPIR.V
6548	▸1	590.0804	2356.2923	2356.2863	2.57	1	32	0.11	▸1	U	R.LFIKPVGDNADTNVTIITNRR.V
7050		812.8027	4058.9769	4058.8909	21.2	0	28	0.041	▸1	U	R.FVYPAASSVDAASSSDLGGGVSFI
7053	▸3	1015.7635	4059.0247	4058.8909	33.0	0	90	3.2e-07	▸1	U	R.FVYPAASSVDAASSSDLGGGVSFI
7064	▸1	1047.7894	4187.1285	4186.9859	34.0	1	72	1.8e-05	▸1	U	R.FVYPAASSVDAASSSDLGGGVSFI

▸5 subsets and intersections (6 subset proteins in total)

(a)

```
MS data file              : 2.mgf
Database                  : NCBIprot 20160920 (95963614 sequences; 35367425320 residues)
Timestamp                 : 12 Oct 2016 at 22:54:09 GMT
Enzyme                    : Trypsin
Fixed modifications       : Carbamidomethyl (C)
Variable modifications    : Oxidation (M)
Mass values               : Monoisotopic
Protein Mass              : Unrestricted
Peptide Mass Tolerance    : ± 1.2 Da
Fragment Mass Tolerance   : ± 0.6 Da
Max Missed Cleavages      : 2
Instrument type           : ESI-QUAD
Number of queries         : 12
Protein hits              : ACM49812.1 VirB10 protein (virB10) [Anaplasma marginale str. Florida]
```

Select Summary Report

Format As | Select Summary (protein hits) | Help

Significance threshold p< 0.05 Max. number of hits 1

Standard scoring ◉ MudPIT scoring ○ Ions score or expect cut-off 0 Show sub-sets 0

Show pop-ups ○ Suppress pop-ups ◉ Require bold red ☐

Preferred taxonomy All entries

Re-Search ◉ All queries ○ Unassigned ○ Below homology threshold ○ Below identity threshold

1. ACM49812.1 **Mass:** 48041 **Score:** 102 **Matches:** 3(0) **Sequences:** 3(0)

VirB10 protein (virB10) [Anaplasma marginale str. Florida]

Query	Observed	Mr(expt)	Mr(calc)	Delta	Miss	Score	Expect	Rank	Unique	Peptide
4	1334.1390	1333.1317	1332.7190	0.4126	0	33	2.1e+02	1	U	K.VFVNQDIVFPR.E
7	1662.2818	1661.2745	1660.7845	0.4900	0	41	29	2	U	R.LIGSYFFDASGNNTR.V
9	2218.7961	2217.7888	2217.1753	0.6135	0	28	5.2e+02	1	U	R.VILPHGIDIQINSAGTD

Proteins matching the same set of peptides:

AGZ80060.1 **Mass:** 47957 **Score:** 102 **Matches:** 3(0) **Sequences:** 3(0)
hypothetical protein U370_04935 [Anaplasma marginale str. Dawn]

AAV87106.1 **Mass:** 48072 **Score:** 102 **Matches:** 3(0) **Sequences:** 3(0)
VirB10 protein [Anaplasma marginale str. St. Maries]

WP_010266574.1 **Mass:** 47652 **Score:** 102 **Matches:** 3(0) **Sequences:** 3(0)
hypothetical protein [Anaplasma marginale]

WP_012880283.1 **Mass:** 48102 **Score:** 102 **Matches:** 3(0) **Sequences:** 3(0)
hypothetical protein [Anaplasma centrale]

WP_037348700.1 **Mass:** 47683 **Score:** 102 **Matches:** 3(0) **Sequences:** 3(0)
hypothetical protein [Anaplasma marginale]

WP_041641675.1 **Mass:** 47568 **Score:** 102 **Matches:** 3(0) **Sequences:** 3(0)
hypothetical protein [Anaplasma marginale]

(b)

Figure S1. (**a**) Mass spec data output for VirB9-1; (**b**) Mass spec data output for VirB10.

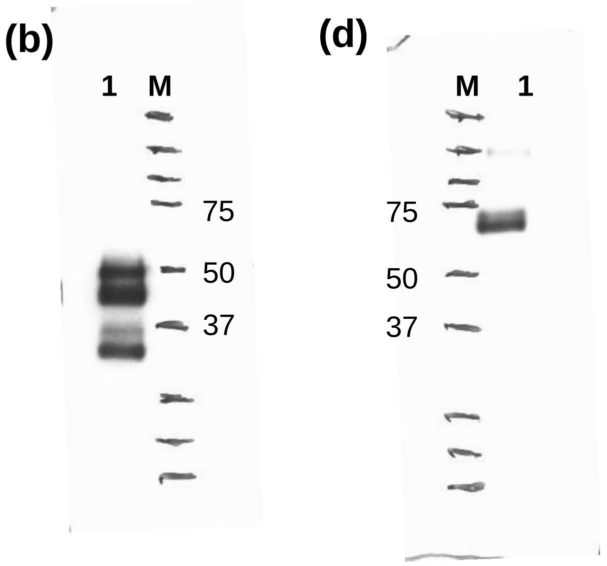

Figure S2. Full image of Western Blot of Figure 1**b**,**d**, respectively.

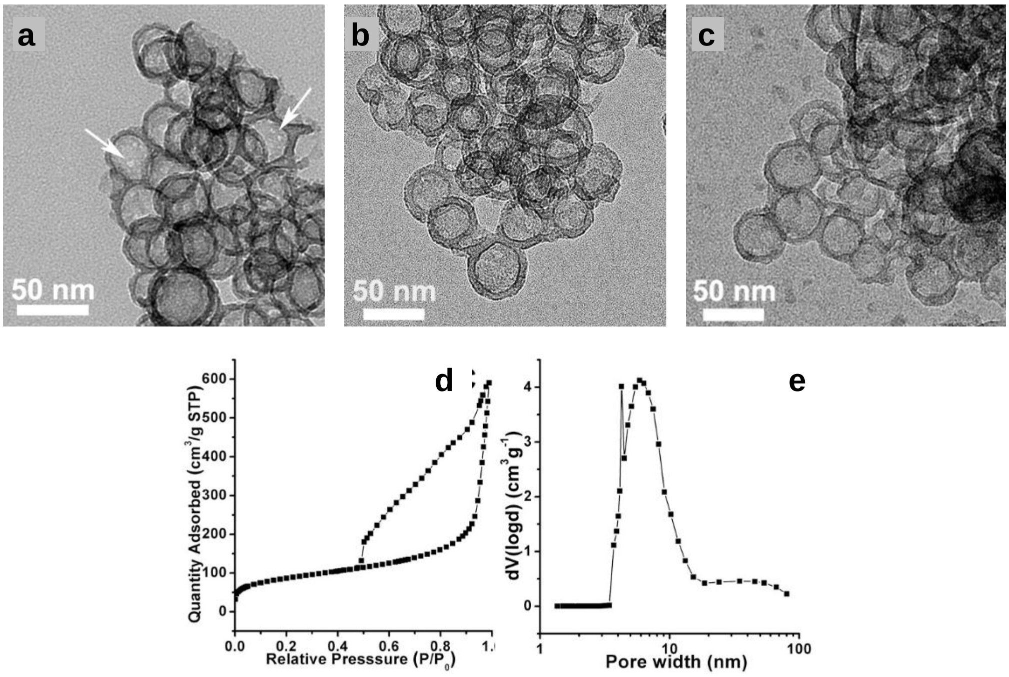

Figure S3. TEM images of SV-100 (**a**) before and after the loading of VirB9-1 (**b**) and VirB10 (**c**) antigens; (**d**) nitrogen sorption isotherm plot and (**e**) BJH pore size distribution curve calculated from desorption branch of SV-100.

SV-100 nanoparticles have a hollow spherical structure with an average particle size of ~50 nm, a wall thickness of ~6 nm and a cavity size of ~40 nm, as measured from the transmission electron microscopy (TEM) image (Figure S3a). Small openings (entrances) on the siliceous walls can be directly observed (indicated by white arrows) with the size of <10 nm. After the adsorption of VirB9-1 and VirB10 antigens, the morphology of SV-100 is maintained (Figure S3b,S3c). It is difficult to see the loaded proteins possibly due to the TEM sample preparation process (dispersed in ethanol solution with sonication). In the nitrogen sorption analysis, SV-100 nanoparticles show type IV isotherms with type H3 hysteresis loop (Figure S3d). The adsorption branch shows major capillary condensation step at relative pressure (P/P_0) of ~0.9. The average entrance size calculated by the Barrett-Joyner-Halanda (BJH) method from the desorption branch was 5.9 nm (Figure S3e), which is in accordance with the observation from the TEM image and also suitable for VirB9-1 and VirB10

antigen loading. The Brunauer-Emmett-Teller (BET) surface area of SV-100 is 491 m^2/g, the total pore volume is 1.39 cm^3/g, and the Zeta potential is −18.5 mV.

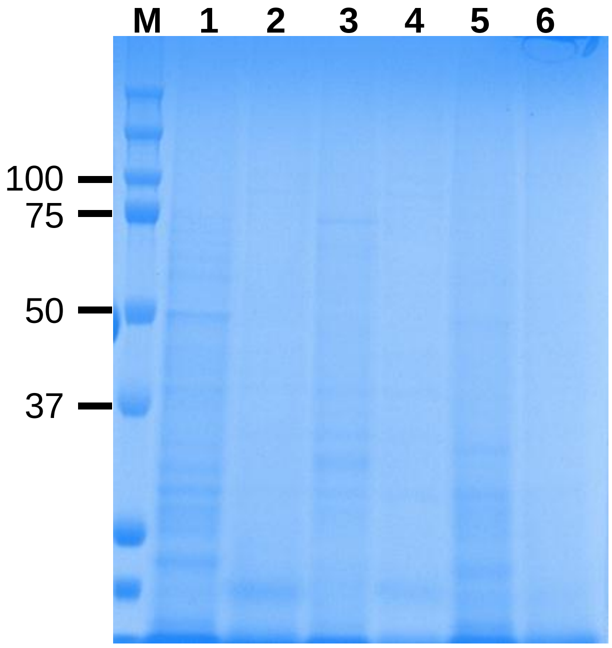

Figure S4. Stability of protein adsorbed SV-100 at 2 months. VirB9-1 and VirB10 proteins adsorbed onto SV-100 particles were stored at 4 °C and room temperature for 2 months. Protein adsorbed SV-100 were run on an SDS-PAGE gel to determined stability. Lane 1 = VirB10 + SV−100 pellets 19.5 µg at 4 °C; Lane 2 = VirB10 19.5 µg at 4 °C; Lane 3 = VirB9-1 + SV-100 9 µg at 4 °C; Lane 4 = VirB9-1 9 µg at 4 °C; Lane 5 = VirB10 + SV-100 pellets from 30 µL = 19.5 µg at RT; Lane 6 = VirB10 19.5 µg at RT.

Table S1a. ELISA dilution data for VirB9-1-specific antibody total IgG level.

VirB9-1_Q					VirB9-1_SV				
**Dilution**	**Mouse 1**	**Mouse 2**	**Mouse 3**	**Mouse 4**	**Dilution**	**Mouse 1**	**Mouse 2**	**Mouse 3**	**Mouse 4**
	**OD450 nm**					**OD450 nm**			
**100**	2.255	2.088	2.022	2.266	**100**	2.829	2.452	2.394	2.261
**200**	2.241	1.828	1.797	2.115	**200**	2.836	2.576	2.422	2.314
**400**	2.195	1.547	1.382	1.908	**400**	2.644	2.475	2.297	2.176
**800**	2.056	1.117	0.995	1.618	**800**	2.385	2.431	2.082	1.652
**1600**	1.858	0.706	0.622	1.288	**1600**	2.374	2.323	1.789	1.669
**3200**	1.519	0.394	0.311	0.886	**3200**	2.114	2.182	1.367	1.217
**6400**	1.150	0.242	0.188	0.588	**6400**	1.868	1.809	0.980	0.917
**VirB9-1/B10_Q**					**VirB9-1/B10_SV**				
**Dilution**	**Mouse 1**	**Mouse 2**	**Mouse 3**	**Mouse 4**	**Dilution**	**Mouse 1**	**Mouse 2**	**Mouse 3**	**Mouse 4**
	**OD450 nm**					**OD450 nm**			
**100**	2.059	2.092	2.210	2.337	**100**	2.405	2.080	2.281	2.307
**200**	1.944	1.948	2.173	2.230	**200**	2.337	2.189	2.309	2.320
**400**	1.932	1.763	2.209	2.032	**400**	2.261	2.223	2.275	2.322
**800**	1.778	1.521	2.124	1.838	**800**	2.225	2.144	2.333	2.212
**1600**	1.502	1.147	1.940	1.250	**1600**	1.999	2.147	2.274	1.888
**3200**	1.017	0.758	1.571	1.069	**3200**	1.670	2.017	2.162	1.615

**6400**	0.643	0.436	1.209	0.698	**6400**	1.322	1.783	2.077	1.171
**SV-100**					**Unimmunised**				
**Dilution**	**Mouse 1**	**Mouse 2**	**Mouse 3**	**Mouse 4**	**Dilution**	**Mouse 1**	**Mouse 2**	**Mouse 3**	**Mouse 4**
	**OD450 nm**					**OD450 nm**			
**100**	0.027	0.010	0.033	0.028	**100**	0.031	0.015	0.021	0.018
**200**	0.024	0.016	0.021	0.013	**200**	0.013	0.003	0.006	0.011
**400**	0.008	0.002	0.011	0.010	**400**	0.005	0.000	0.002	0.002
**800**	0.015	0.000	0.006	0.004	**800**	−0.001	−0.002	−0.005	0.000
**1600**	0.011	−0.001	0.001	0.009	**1600**	−0.001	−0.003	−0.007	−0.004
**3200**	0.004	0.003	0.050	0.009	**3200**	−0.004	−0.009	−0.003	−0.004
**6400**	0.001	−0.001	−0.002	0.018	**6400**	−0.006	−0.007	−0.005	0.000

Table S1b. ELISA dilution data for VirB10-specific antibody total IgG level.

**Group 2 VirB10_Q**					**VirB10_SV**				
**Dilution**	**Mouse 1**	**Mouse 2**	**Mouse 3**	**Mouse 4**	**Dilution**	**Mouse 1**	**Mouse 2**	**Mouse 3**	**Mouse 4**
	**OD450 nm**					**OD450 nm**			
**100**	1.597	1.974	1.738	1.902	**100**	2.747	2.992	2.323	2.790
**200**	1.779	2.170	2.053	2.088	**200**	2.735	3.018	2.415	2.817
**400**	1.899	2.249	2.251	2.229	**400**	2.640	3.208	2.569	2.949
**800**	1.983	2.281	1.887	2.040	**800**	2.231	3.227	2.580	2.952
**1600**	1.888	2.163	1.703	1.729	**1600**	1.799	3.310	2.528	2.903
**3200**	1.639	1.822	1.324	1.299	**3200**	1.212	3.244	2.575	2.503
**6400**	1.287	1.493	0.915	0.861	**6400**	0.761	2.907	2.351	2.053
**VirB9-1/B10_Q**					**VirB9-1/B10-SV**				
**Dilution**	**Mouse 1**	**Mouse 2**	**Mouse 3**	**Mouse 4**	**Dilution**	**Mouse 1**	**Mouse 2**	**Mouse 3**	**Mouse 4**
	**OD450 nm**					**OD450 nm**			
**100**	1.751	2.020	1.780	2.334	**100**	2.068	1.740	2.046	2.319
**200**	1.740	2.074	1.727	2.270	**200**	2.211	1.794	2.112	2.502
**400**	1.820	2.025	1.827	2.343	**400**	2.318	1.823	2.231	2.230
**800**	1.859	2.094	2.002	2.196	**800**	2.498	2.074	2.412	2.627
**1600**	1.755	1.974	1.908	2.107	**1600**	2.447	2.087	2.486	2.610
**3200**	1.589	1.660	1.720	1.795	**3200**	1.975	1.917	2.429	2.567
**6400**	1.350	1.412	1.423	1.397	**6400**	1.503	1.591	2.185	2.217
**SV-100**					**Unimmunised**				
**Dilution**	**Mouse 1**	**Mouse 2**	**Mouse 3**	**Mouse 4**	**Dilution**	**Mouse 1**	**Mouse 2**	**Mouse 3**	**Mouse 4**
	**OD450 nm**					**OD450 nm**			
**100**	0.023	0.006	0.026	0.019	**100**	0.010	0.004	0.014	0.005
**200**	0.012	0.004	0.016	0.007	**200**	0.002	0.000	0.003	0.001
**400**	0.004	−0.002	0.011	0.011	**400**	−0.002	−0.004	−0.001	−0.006
**800**	0.003	−0.002	0.007	0.000	**800**	−0.005	−0.001	−0.003	−0.002

**1600**	0.000	0.000	0.001	0.009	**1600**	−0.006	−0.003	−0.001	−0.003
**3200**	0.001	−0.003	0.006	0.049	**3200**	−0.003	−0.003	−0.005	−0.004
**6400**	0.001	−0.003	0.002	−0.002	**6400**	−0.004	−0.004	−0.004	−0.005

© 2016 by the authors. Submitted for possible open access publication under the terms and conditions of the Creative Commons Attribution (CC-BY) license (http://creativecommons.org/licenses/by/4.0/).
